# Posterior Reversible Encephalopathy Syndrome in Eclampsia: A Prospective Study of Risk Factors and Outcomes

**DOI:** 10.7759/cureus.110179

**Published:** 2026-06-03

**Authors:** Preeti Banerjee, Pooja Sahu, Zoya Naaz, Aneesa Mahmood, Nazma Begum, Chowdavarapu R Rao

**Affiliations:** 1 Department of Obstetrics and Gynecology, Deccan College of Medical Sciences, Hyderabad, IND; 2 Department of Obstetrics and Gynecology, Apollo Institute of Medical Sciences and Research, Hyderabad, IND; 3 Department of Maternal and Child Health Nutrition, ICMR-National Institute of Nutrition, Hyderabad, IND

**Keywords:** eclampsia, maternal outcomes, neonatal outcomes, posterior reversible encephalopathy syndrome, risk factors

## Abstract

Introduction

Posterior reversible encephalopathy syndrome is an important neurological complication associated with eclampsia that contributes significantly to maternal and perinatal morbidity in high-risk pregnancies. This study aimed to determine the prevalence of posterior reversible encephalopathy syndrome in women with eclampsia and to evaluate the associated risk factors, radiological findings, and maternal and perinatal outcomes.

Materials and methods

This prospective observational study was conducted at a tertiary care teaching hospital in Hyderabad, India, between January 2022 and December 2024. In total, 140 women with eclampsia were enrolled in this study. The diagnosis of posterior reversible encephalopathy syndrome was confirmed using magnetic resonance imaging performed within 48-72 hours of admission. Clinical, laboratory, and imaging data were recorded. Statistical analysis was performed using SPSS Statistics (version 26.0; IBM Inc., Armonk, New York, United States). Continuous variables were compared using the independent samples t-test or Mann-Whitney U test, and categorical variables were compared using the chi-square test or Fisher's exact test. Logistic regression analysis was used to identify the independent predictors.

Results

Posterior reversible encephalopathy syndrome was observed in 62 (44.3%) women. The syndrome was associated with higher systolic blood pressure (178.4 ± 18.6 vs. 162.3 ± 16.2 mmHg; p < 0.001), higher frequency of three or more convulsions (p < 0.001), delayed treatment (p = 0.002), thrombocytopenia, and elevated serum creatinine (p < 0.001). The independent predictors included systolic blood pressure of 170 mmHg or more (adjusted odds ratio 3.21; p = 0.003), three or more convulsions (adjusted odds ratio 3.68; p < 0.001), delayed treatment (adjusted odds ratio 2.54; p = 0.017), platelet count less than 100 × 10³ per microliter (adjusted odds ratio 2.24; p = 0.044), and serum creatinine of 1.2 mg/dL or more (adjusted odds ratio 2.86; p = 0.008). Maternal complications, including intensive care unit admission (p < 0.001) and acute kidney injury (p < 0.001), were higher in the posterior reversible encephalopathy syndrome group. Neonatal outcomes were also worse, with higher rates of low birth weight (p = 0.001) and preterm birth (p < 0.001).

Conclusion

Posterior reversible encephalopathy syndrome is a frequent complication of eclampsia and is associated with a severe clinical presentation and adverse maternal and neonatal outcomes. Early identification and timely management of high-risk patients are essential for improving prognosis.

## Introduction

Eclampsia remains a major cause of maternal and perinatal morbidity and mortality, particularly in developing countries, with a prevalence rate of 1.8% to 16.7%, where access to timely antenatal care and emergency obstetric services may be limited [1]. It is characterized by the occurrence of new-onset generalized tonic-clonic seizures in women with preeclampsia and is frequently associated with severe systemic and neurological complications [2]. Among these, posterior reversible encephalopathy syndrome (PRES) has emerged as a critical neuroradiological entity, characterized by vasogenic edema, predominantly affecting the posterior regions of the brain [3]. Early recognition of PRES is essential because timely intervention can lead to complete neurological recovery, whereas delayed management may result in irreversible brain injury or death.

The pathophysiology of PRES in eclampsia is complex and not yet fully understood, involving endothelial dysfunction, failure of cerebral autoregulation, and abrupt elevations in blood pressure, leading to blood-brain barrier disruption [4]. Magnetic resonance imaging (MRI), particularly with T2-weighted and FLAIR sequences, plays a pivotal role in confirming the diagnosis and distinguishing vasogenic edema from cytotoxic edema [5]. Despite increasing recognition, the true incidence of PRES among women with eclampsia and its associated clinical, laboratory, and radiological predictors remains inadequately characterized, especially in resource-limited settings [6].

Furthermore, the presence of PRES has been linked to worse maternal and perinatal outcomes, including an increased need for intensive care, higher rates of complications, and adverse neonatal events [4]. However, prospective studies evaluating these associations are limited. This study aimed to determine the prevalence of PRES among women with eclampsia and evaluate the associated risk factors, radiological characteristics, and maternal and perinatal outcomes. The specific objectives were to identify independent predictors of PRES using logistic regression analysis, assess clinical and biochemical correlates, evaluate maternal outcomes, including neurological recovery, and analyze fetal and neonatal outcomes in affected patients.

## Materials and methods

This prospective observational study was conducted in the Department of Obstetrics and Gynecology at the Deccan College of Medical Sciences, Kanchanbagh, Hyderabad, Telangana, India, in collaboration with the Department of Radiodiagnosis and Neurology. The study was carried out over a period of three years, from January 2022 to December 2024. Ethical approval was obtained from the Institutional Ethics Committee of Deccan College of Medical Sciences (2022/36/008) prior to initiation of the study, and all procedures were conducted in accordance with the Declaration of Helsinki. Written informed consent was obtained from all participants or their legally authorized representatives before enrolment.

The study employed consecutive sampling, wherein all eligible women with eclampsia who met the inclusion criteria during the study period were prospectively enrolled. All pregnant women and women in the immediate postpartum period, within 48 hours of delivery, who were diagnosed with eclampsia were prospectively included. Eclampsia was defined as new-onset generalized tonic-clonic seizures in a woman with preeclampsia, in the absence of other neurological causes. The inclusion criteria were gestational age of 20 weeks or more or within 48 hours postpartum, confirmed diagnosis of eclampsia, and willingness to participate in follow-up compliance. The exclusion criteria included prior seizure disorders, epilepsy, structural brain lesions such as intracranial hemorrhage, cerebral venous thrombosis, brain tumors, metabolic causes of seizures, incomplete clinical records, and patients lost to follow-up.

The sample size was estimated based on a reported prevalence of PRES of 16% with a confidence level of 95% and a margin of error of 6.4%, yielding a minimum sample size of 126 [4]. Considering a possible attrition rate of approximately 10%, 140 women with eclampsia were enrolled in this study.

Sociodemographic variables included age, gravidity, gestational age at the onset of eclampsia, and adequacy of antenatal care, defined as four or more antenatal visits. Clinical parameters recorded at admission included systolic and diastolic blood pressure, number of convulsions, time interval from seizure onset to treatment initiation, and neurological status. All patients received standardized management according to the institutional protocols, including intravenous magnesium sulfate for seizure control and antihypertensive therapy for blood pressure management.

Laboratory investigations performed at admission included complete blood count with platelet count, serum creatinine, and liver function tests, including alanine aminotransferase, aspartate aminotransferase, serum uric acid, and lactate dehydrogenase. Acute kidney injury was diagnosed based on elevated serum creatinine and clinical assessment according to institutional protocols. Neuroimaging was performed using a 1.5 Tesla magnetic resonance imaging system (GE Healthcare, Chicago, Illinois, USA) with standardized sequences including T2-weighted imaging, fluid-attenuated inversion recovery, diffusion-weighted imaging, and apparent diffusion coefficient mapping. These sequences were used to identify vasogenic edema and confirm the diagnosis of PRES. MRI was performed within 48-72 hours of admission. All scans were interpreted by an experienced radiologist. Owing to limitations in image archival and retrieval, representative MRI images were not available in a format suitable for publication.

The primary outcome was the incidence of PRES in women with eclampsia, as confirmed by the MRI findings [7]. Secondary outcomes included identification of independent risk factors; maternal outcomes such as ICU admission, duration of hospital stay, need for mechanical ventilation, complications, and neurological recovery; and perinatal outcomes including birth weight, preterm birth, Apgar score at five minutes, neonatal intensive care unit admission, and mortality. Complete neurological recovery was defined as resolution of acute neurological symptoms without persistent focal neurological deficits at discharge. MRI scans were interpreted by experienced radiologists as part of routine clinical practice; however, blinded independent review and interobserver agreement analysis were not performed.

All collected data were entered into Microsoft Excel (Microsoft Corporation, Redmond, Washington, USA) and analyzed using SPSS Statistics for Windows, version 26.0 (IBM Inc., Armonk, New York, USA). Continuous variables were assessed for normality using the Shapiro-Wilk test. Normally distributed data were expressed as mean ± standard deviation and compared using the independent samples t-test, whereas non-normally distributed data were presented as medians with interquartile ranges and compared using the Mann-Whitney U test. Categorical variables were expressed as frequencies and percentages and compared using the chi-squared test or Fisher's exact test, as appropriate. Univariate logistic regression analysis was performed to identify potential risk factors, followed by multivariate logistic regression analysis, including variables with p < 0.05. Results were expressed as odds ratios with 95% confidence intervals, and a p-value of less than 0.05 was considered statistically significant.

## Results

A total of 140 women with eclampsia were included (Table 1), of which 62 (44.3%) had PRES, and 78 (55.7%) did not. The mean age was comparable between groups (24.3 ± 4.8 vs. 25.1 ± 5.2 years; p = 0.342). Primigravida status was observed in 44 (71.0%) and 51 (65.4%) women in the PRES and non-PRES groups, respectively (p = 0.479). The rate of adequate antenatal care (≥4 visits) was significantly lower in the PRES group (p = 0.018). Clinical severity was higher in the PRES group, with significantly elevated systolic and diastolic blood pressures (178.4 ± 18.6 vs. 162.3 ± 16.2 mmHg and 116.2 ± 12.4 vs. 106.8 ± 11.7 mmHg; both p < 0.001). A higher proportion of women in the PRES group experienced ≥3 convulsions (p < 0.001), and delayed treatment was also observed (median 6.2 vs. 3.4 hours; p = 0.002). Laboratory abnormalities were more frequent in the PRES group, including a platelet count <100×10³/μL, elevated serum creatinine, elevated lactate dehydrogenase (LDH), and elevated liver enzymes (p = 0.005). The number of antenatal visits was recorded for all participants, and adequate antenatal care was defined as four or more antenatal visits during pregnancy.

**Table 1:** Baseline demographic and clinical characteristics of study participants Values are presented as n (%), mean ± standard deviation (SD), or median (interquartile range). Independent samples t-test was used for normally distributed continuous variables, Mann-Whitney U test for non-normally distributed variables, and chi-squared test for categorical variables. *p-value < 0.05 was considered statistically significant. BP - blood pressure; ALT - alanine aminotransferase; AST - aspartate aminotransferase; ULN - upper limit of normal; LDH - lactate dehydrogenase; IQR - interquartile range; PRES - posterior reversible encephalopathy syndrome

Variable	PRES group (n = 62)	Non-PRES group (n = 78)	Test value	p-value
Sociodemographic characteristics				
Age (years), mean ± SD	24.3 ± 4.8	25.1 ± 5.2	t = 0.94	0.342
Primigravida, n (%)	44 (71.0)	51 (65.4)	χ² = 0.49	0.479
Gestational age at onset (weeks), mean ± SD	33.2 ± 3.6	35.4 ± 3.1	t = 3.82	0.001*
Antenatal care (≥4 visits), n (%)	18 (29.0)	38 (48.7)	χ² = 5.58	0.018*
Clinical characteristics at presentation				
Systolic BP (mmHg), mean ± SD	178.4 ± 18.6	162.3 ± 16.2	t = 5.38	< 0.001*
Diastolic BP (mmHg), mean ± SD	116.2 ± 12.4	106.8 ± 11.7	t = 4.57	< 0.001*
Proteinuria (≥3+), n (%)	54 (87.1)	58 (74.4)	χ² = 3.50	0.063
Number of convulsions (≥3), n (%)	38 (61.3)	22 (28.2)	χ² = 15.44	< 0.001*
Time to treatment (hours), median (IQR)	6.2 (3.8–9.4)	3.4 (2.1–5.8)	U = 987	0.002*
Laboratory parameters				
Serum creatinine (mg/dL), mean ± SD	1.42 ± 0.58	1.08 ± 0.41	t = 3.90	< 0.001*
Platelet count (×10³/μL), mean ± SD	98.4 ± 42.6	142.7 ± 56.3	t = 5.30	< 0.001*
ALT/AST elevated (>2× ULN), n (%)	31 (50.0)	21 (26.9)	χ² = 7.88	0.005*
Serum uric acid (mg/dL), mean ± SD	7.8 ± 1.6	6.4 ± 1.4	t = 5.43	< 0.001*
LDH (U/L), mean ± SD	648.2 ± 214.8	428.6 ± 168.4	t = 6.60	< 0.001*

Univariate analysis identified several significant predictors of PRES (Table 2). On multivariate logistic regression, independent predictors included systolic blood pressure ≥170 mmHg, ≥3 convulsions, delayed treatment >4 h, platelet count <100×10³/μL, and serum creatinine ≥1.2 mg/dL (all p < 0.05).

**Table 2:** Logistic regression analysis of risk factors for posterior reversible encephalopathy syndrome in women with eclampsia Odds ratios were calculated using logistic regression analysis. Variables with p < 0.05 in univariate analysis were included in multivariate logistic regression. Results are expressed as odds ratios with 95% confidence interval. *p-value < 0.05 was considered statistically significant. OR - odds ratio; CI - confidence interval; ALT - alanine aminotransferase; AST - aspartate aminotransferase; ULN - upper limit of normal; LDH - lactate dehydrogenase

Variable	Univariate OR (95% CI)	p-value	Adjusted OR (95% CI)	p-value
Systolic BP ≥170 mmHg	3.84 (1.92–7.68)	0.001*	3.21 (1.48–6.96)	0.003*
Number of convulsions ≥3	4.12 (2.06–8.24)	<0.001*	3.68 (1.74–7.78)	< 0.001*
Delayed treatment (>4 hrs)	2.96 (1.48–5.92)	0.002*	2.54 (1.18–5.46)	0.017*
Platelet count <100×10³/μL	2.68 (1.34–5.36)	0.005*	2.24 (1.02–4.92)	0.044*
Serum creatinine ≥1.2 mg/dL	3.14 (1.57–6.28)	0.001*	2.86 (1.32–6.18)	0.008*
LDH ≥600 U/L	2.42 (1.21–4.84)	0.012*	1.98 (0.88–4.44)	0.098
Gestational age <34 weeks	2.18 (1.09–4.36)	0.027*	1.84 (0.82–4.12)	0.138
Inadequate ANC (<4 visits)	2.36 (1.18–4.72)	0.015*	1.76 (0.78–3.96)	0.172
Elevated ALT/AST (>2× ULN)	2.72 (1.36–5.44)	0.004*	2.12 (0.96–4.68)	0.062

Maternal outcomes were summarized in Table 3. ICU admission was required in 48 (77.4%) women in the PRES group compared with 22 (28.2%) in the non-PRES group (p < 0.001). Mechanical ventilation was needed in 18 (29.0%) and four (5.1%) patients, respectively (p < 0.001). Acute kidney injury occurred in 22 (35.5%) vs. eight (10.3%) patients (p < 0.001), pulmonary edema in 12 (19.4%) vs. five (6.4%) patients (p = 0.018), and visual disturbances or cortical blindness in 26 (41.9%) vs. six (7.7%) patients (p < 0.001). Complete neurological recovery was achieved in 52 (83.9%) patients with PRES. Maternal mortality was higher in the PRES group (four (6.5%) vs. one (1.3%)); however, this difference was not statistically significant (p = 0.117).

**Table 3:** Maternal outcomes in posterior reversible encephalopathy syndrome (PRES) and non-posterior reversible encephalopathy syndrome groups Values are presented as n (%), mean ± standard deviation, or median (interquartile range). Independent samples t-test, Mann–Whitney U test, chi-squared test, and Fisher's exact test were used as appropriate. *p-value < 0.05 was considered statistically significant. IQR - interquartile range; ICU - intensive care unit

Parameter	PRES group (n = 62)	Non-PRES group (n = 78)	Test value	p-value
ICU admission, n (%)	48 (77.4)	22 (28.2)	χ² = 33.47	< 0.001*
Duration of ICU stay (days), median (IQR)	5.4 (3.0–8.2)	2.1 (1.0–3.6)	U= 1243	< 0.001*
Duration of hospital stay (days), mean ± SD	12.6 ± 5.8	7.4 ± 3.2	t = 6.33	< 0.001*
Mechanical ventilation, n (%)	18 (29.0)	4 (5.1)	χ² = 14.91	< 0.001*
Acute kidney injury, n (%)	22 (35.5)	8 (10.3)	χ² = 13.06	< 0.001*
Pulmonary edema, n (%)	12 (19.4)	5 (6.4)	χ² = 5.43	0.018*
Visual disturbances/cortical blindness, n (%)	26 (41.9)	6 (7.7)	χ² = 22.97	< 0.001*
Complete neurological recovery, n (%)	52 (83.9)	0 (0.0)	Not applicable	Not applicable
Maternal mortality, n (%)	4 (6.5)	1 (1.3)	Fisher’s exact OR = 5.31	0.117

Cesarean section was more common in the PRES group (Table 4), with emergency procedures accounting for 42 (87.5%) and 30 (65.2%) cases, respectively (p = 0.012). Vaginal delivery was less frequent (p = 0.021). Neonatal outcomes were significantly worse in the PRES group, including low birth weight (p = 0.001), preterm birth (p < 0.001), low Apgar score at five minutes (p = 0.001), neonatal intensive care unit admission (p < 0.001), and respiratory distress syndrome (p = 0.002). Intrauterine fetal death (p = 0.184) and early neonatal death (p = 0.148) were not statistically significant, whereas perinatal mortality was significantly higher (p = 0.028).

**Table 4:** Mode of delivery and fetal and neonatal outcomes in posterior reversible encephalopathy syndrome (PRES) and non-posterior reversible encephalopathy syndrome groups Perinatal mortality rate calculated per 1000 births, including stillbirths and early neonatal deaths (within seven days of birth). Values are presented as n (%), mean ± standard deviation, or median (interquartile range). Independent samples t-test, Mann–Whitney U test, chi-squared test, and Fisher's exact test were used as appropriate. *p-value < 0.05 was considered statistically significant. IQR - interquartile range; CS - cesarean section; NICU - neonatal intensive care unit; RDS - respiratory distress syndrome

Parameters	Outcome	PRES group (n = 62)	Non-PRES group (n = 78)	Test value	p-value
Mode of delivery	Vaginal delivery, n (%)	14 (22.6)	32 (41.0)	χ² = 5.327	0.021*
	Cesarean section (total), n (%)	48 (77.4)	46 (59.0)		
Caesarean section	Emergency CS, n (%)	42 (87.5)	30 (65.2)	χ² = 6.505	0.012*
	Elective CS, n (%)	6 (12.5)	16 (34.8)		
Fetal / neonatal outcomes	Birth weight (grams), mean ± SD	2,048 ± 46	2,412 ± 51	t = 4.387	<0.001*
	Low birth weight (<2500 g), n (%)	48 (77.4)	38 (48.7)	χ² = 12.009	0.001*
	Preterm birth (<37 weeks), n (%)	52 (83.9)	42 (53.8)	χ² = 14.116	<0.001*
	Apgar score at 5 min (≤6), n (%)	28 (45.2)	14 (17.9)	χ² = 12.181	0.001*
	NICU admission, n (%)	44 (71.0)	28 (35.9)	χ² = 17.010	<0.001*
	NICU stay (days), median (IQR)	12.4 (7.0–18.6)	6.8 (3.2–11.4)	U ≈ 3126	0.003*
	RDS requiring surfactant, n (%)	22 (35.5)	10 (12.8)	χ² = 10.061	0.002*
	Intrauterine fetal death (IUFD), n (%)	6 (9.7)	3 (3.8)	Fisher’s exact: OR = 2.68	0.184
	Early neonatal death, n (%)	5 (8.1)	2 (2.6)	Fisher’s exact: OR = 3.33	0.148
	Perinatal mortality rate (per 1000 births)	177.4	64.1	χ² = 4.382	0.028*

**Figure 1:**
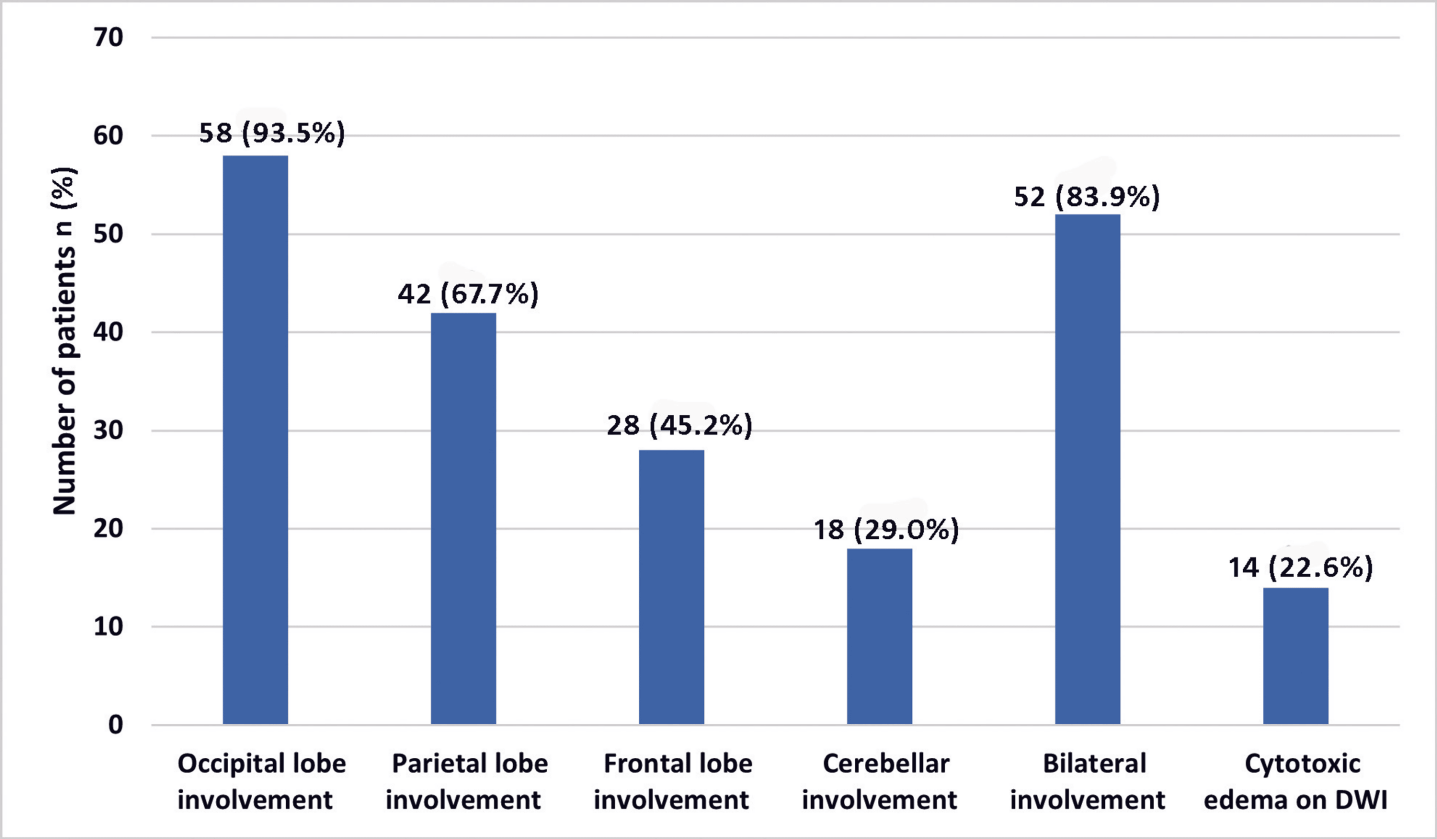
Magnetic resonance imaging findings in patients with posterior reversible encephalopathy syndrome Bar diagram showing the distribution of brain involvement in patients with posterior reversible encephalopathy syndrome. Data presented as n (%). DWI - diffusion-weighted imaging

Figure 1 demonstrates MRI findings in patients with PRES. Occipital lobe involvement was the most common finding, observed in 58 (93.5%) patients, followed by bilateral involvement in 52 (83.9%), and parietal lobe involvement in 42 (67.7%). Frontal lobe involvement was observed in 28 (45.2%) patients, and cerebellar involvement in 18 (29.0%) patients. Cytotoxic edema on diffusion-weighted imaging was present in 14 (22.6%) patients, indicating a predominantly posterior and bilateral pattern of involvement.

## Discussion

The present prospective study evaluated the incidence, risk factors, and outcomes of PRES among women with eclampsia and demonstrated that PRES is a frequent and clinically significant complication. This relatively high incidence aligns with a previous study that reported a wide prevalence range, reflecting differences in diagnostic criteria and imaging availability [1]. Studies similarly highlighted the strong association between hypertensive disorders of pregnancy and PRES, emphasizing the importance of early recognition [7,8].

In the present study, patients with PRES exhibited more severe clinical profiles at presentation, including significantly higher blood pressure, increased frequency of convulsions, and delayed initiation of treatment. These findings are consistent with the pathophysiological basis of PRES, wherein an abrupt elevation in blood pressure overwhelms cerebral autoregulation, leading to endothelial dysfunction and vasogenic edema [9]. A previous study by Bartynski [10] demonstrated that severe hypertension and recurrent seizures are key contributors to cerebral edema formation in eclampsia-related PRES.

Multivariate analysis in this study identified systolic blood pressure ≥170 mmHg, ≥3 convulsions, delayed treatment, thrombocytopenia, and elevated serum creatinine level as independent predictors of PRES. A retrospective analysis by Bahadur et al. [6] identified severe hypertension, recurrent seizures, and delayed treatment as significant predictors of PRES in women with preeclampsia/eclampsia. These findings are consistent with those of the present study, reinforcing the role of clinical severity and timely intervention in determining the risk of PRES. Elevated serum creatinine and low platelet counts may reflect underlying renal impairment and microangiopathic processes, which are known contributors to disease severity in preeclampsia and eclampsia. Similar associations were reported by Lee et al. [11] and Shaikh et al. [4], reinforcing the role of these laboratory markers as predictors of adverse neurological outcomes.

Maternal outcomes in the present study were significantly worse in patients with PRES, with higher rates of intensive care unit admission, prolonged hospital stay, mechanical ventilation, and complications, such as acute kidney injury, pulmonary edema, and visual disturbances. These findings corroborate earlier studies demonstrating increased morbidity associated with PRES [12]. Although most patients achieve complete neurological recovery, the higher complication burden underscores the need for early identification and aggressive management. The observed maternal mortality, although not statistically significant, was higher in the PRES group, consistent with previous literature suggesting an increased risk in severe cases [12,13].

Neonatal outcomes were also adversely affected in the PRES group, with significantly higher rates of low birth weight, preterm birth, low Apgar score, and respiratory distress syndrome. These findings likely reflect both the severity of the maternal disease and the increased likelihood of early or emergency delivery. Previous studies have similarly reported poorer perinatal outcomes in women with severe hypertensive disorders complicated by PRES, indicating that fetal compromise is closely linked to maternal hemodynamic instability and placental insufficiency [14,15].

Radiologically, the predominance of occipital and bilateral involvement observed in this study is consistent with the classical description of PRES. Posterior circulation is particularly vulnerable due to relatively reduced sympathetic innervation, making it more susceptible to autoregulatory failure. The presence of cytotoxic edema in a subset of patients suggests that delayed diagnosis or severe disease may lead to irreversible injury, further highlighting the importance of early imaging [5,16].

The clinical implications of this study are significant. Identification of key risk factors, such as severe hypertension, recurrent seizures, delayed treatment, thrombocytopenia, and renal dysfunction, can facilitate early risk stratification in patients with eclampsia. Prompt neuroimaging in high-risk individuals, along with aggressive blood pressure control and seizure management, may help prevent the progression to severe PRES and improve maternal and neonatal outcomes. Furthermore, strengthening antenatal care services may aid in the early detection and management of preeclampsia, thereby reducing the incidence of eclampsia and complications.

However, this study had certain limitations. Although prospective in design, the study was conducted at a single tertiary care center, which may limit the generalizability of the findings. The observational nature of the study permits identification of associations but does not establish causal relationships between PRES and adverse maternal or neonatal outcomes. Some observed neonatal complications may also reflect the overall severity of maternal disease and related obstetric interventions rather than an independent effect of PRES itself. MRI was performed in all patients; however, representative images were not available for inclusion due to archival limitations. MRI interpretation was performed as part of routine clinical practice, and blinded independent review or interobserver agreement analysis was not undertaken. Additionally, long-term neurological outcomes beyond six weeks postpartum were not assessed. The multivariate regression model included a relatively limited number of events per variable, which may affect model stability and should be interpreted cautiously. Despite these limitations, the present study provides clinically relevant evidence regarding the prevalence and associated factors of PRES in women with eclampsia and highlights the importance of early recognition and timely management in high-risk patients.

## Conclusions

Posterior reversible encephalopathy syndrome was frequently observed among women with eclampsia and was associated with more severe clinical presentation, adverse maternal outcomes, and poorer neonatal outcomes. Higher blood pressure, recurrent convulsions, delayed treatment, thrombocytopenia, and elevated serum creatinine showed significant associations with the presence of PRES. The observed neonatal complications may also reflect the overall severity of maternal disease and related obstetric interventions rather than an independent effect of PRES itself. These findings should therefore be interpreted as associative rather than causal. Early recognition and timely management of high-risk patients may help improve clinical outcomes. Further large-scale prospective studies are required to validate these associations and better clarify the temporal and causal relationships between PRES and maternal and neonatal outcomes.
